# Crystallization Control Through Intermediates in Br‐Enriched Mixed‐Halide Wide‐Bandgap Perovskites

**DOI:** 10.1002/smsc.70428

**Published:** 2026-10-22

**Authors:** Taeho Moon

**Affiliations:** ^1^ Department of Materials Science and Engineering Dankook University Cheonan Korea; ^2^ Hydrogen Research Center Dankook University Cheonan Korea

**Keywords:** crystallization intermediates, halide segregation, mixed‐halide perovskites, tandem and indoor photovoltaics, wide bandgap

## Abstract

Br‐enriched wide‐bandgap halide perovskites are essential for tandem and indoor photovoltaic applications due to their tunable bandgaps and high open‐circuit voltages. However, increasing bromide content intensifies compositional instability and halide segregation, leading to voltage losses and long‐term operational degradation. Emerging evidence indicates that such instability is frequently preconditioned during film formation through crystallization intermediates, rather than merely manifesting as light‐induced halide segregation during operation. This review presents an intermediate‐centric framework for understanding crystallization in Br‐rich mixed‐halide perovskites. Crystallization intermediates are defined as emergent states encompassing both coordination‐defined species and structural intermediates (e.g., polytypes), governed by the competitive binding of halides, solvents, and additives to Pb^2+^ centers, thereby regulating nucleation kinetics, halide redistribution, and lattice consolidation. Strong Pb–Br interactions restrict the range of kinetically accessible intermediate configurations, promoting halide‐segregated growth and kinetically frozen compositional gradients that subsequently appear as defect‐rich regions and ion‐migration pathways. Recent advances demonstrate that solvent and additive engineering reshape intermediate accessibility and lifetime, enabling stabilization, synchronization, reconstruction, or even elimination of intermediate states. An intermediate‐centered perspective on crystallization provides a chemically grounded basis for improving halide homogeneity, defect control, and stability in Br‐rich wide‐bandgap perovskite photovoltaics.

## Graphical abstract

The stronger Pb–Br coordination characteristic of Br‐rich wide‐bandgap perovskites reshapes the accessibility and evolution of crystallization intermediates and promotes asynchronous halide crystallization. Solvent‐coordinated adducts, polytypes, and halide‐rich clusters are viewed as emergent states arising from coordination competition among halides, solvents, additives, and A‐site cations. Controlling these intermediate pathways regulates halide redistribution, compositional homogeneity, and stability.

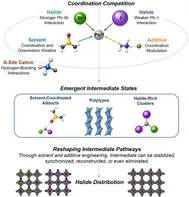

## 1 Introduction

Wide‐bandgap (WBG) semiconductors have emerged as indispensable photoactive materials for next‐generation photovoltaic technologies, particularly in tandem architectures and indoor photovoltaic (IPV) systems [1, 2, 3, 4, 5, 6, 7, 8, 9, 10, 11, 12, 13, 14, 15, 16, 17, 18, 19, 20]. Among the various WBG semiconductor systems investigated for these applications, halide perovskites have attracted particular attention owing to their exceptional optoelectronic properties and compositional tunability. In particular, bandgap widening can be readily achieved through partial substitution of iodide with bromide in ABX_3_ perovskites, enabling continuous bandgap tuning, spectral selectivity, and high open‐circuit voltages (*V*_OC_). However, despite impressive device efficiencies, Br‐enriched mixed‐halide perovskites remain intrinsically prone to compositional instability and halide segregation, which manifest as voltage deficits, enhanced nonradiative recombination, and long‐term operational instability under illumination and thermal stress.

While light‐induced halide segregation is often regarded as the primary degradation pathway in WBG perovskites, increasing evidence indicates that compositional heterogeneity is frequently encoded much earlier—during film formation [21, 22, 23, 24, 25, 26, 27, 28, 29, 30]. In solution‐processed perovskites, crystallization does not proceed directly from homogeneous ionic solutions to a fully ordered three‐dimensional lattice. Instead, it involves a series of transient states, commonly described as crystallization intermediates, encompassing both coordination‐defined species and structural intermediates (e.g., polytypes). These intermediates define the kinetic landscape of nucleation, growth, and lattice consolidation. In Br‐rich systems, where Pb–Br coordination is stronger and structurally more rigid than Pb─I bonding, even subtle variations in precursor chemistry can drastically alter intermediate accessibility, lifetime, and transformation pathways.

Recent advanced characterization techniques, including in situ grazing‐incidence wide‐angle X‐ray scattering (GIWAXS), photoluminescence (PL) spectroscopy, and solution‐state spectroscopy, have revealed that mixed‐halide perovskites frequently crystallize through halide‐segregated pathways. Bromide‐rich fractions tend to nucleate rapidly, whereas iodide‐rich species may transiently persist as solvent‐coordinated or polytype‐related intermediates before incorporation into the perovskite lattice. As a result, compositional inhomogeneities can become kinetically frozen during intermediate collapse. These early‐stage heterogeneities later manifest as defect‐rich regions, local strain fields, and preferential ion‐migration pathways that amplify photoinduced segregation during device operation.

From this perspective, crystallization intermediates should not be regarded merely as transient structural motifs or route‐specific byproducts. Rather, their formation and evolution are governed by the coordination landscape arising from competition among Pb^2+^–halide, Pb^2+^–solvent, and Pb^2+^–additive interactions [31, 32]. In Br‐enriched WBG compositions, this competition becomes increasingly sensitive to precursor chemistry because strong Pb–Br bonding narrows the window of kinetically accessible intermediate configurations. Consequently, the presence, suppression, stabilization, reconstruction, or even elimination of intermediates directly determines halide redistribution, defect formation, and long‐term compositional stability.

Although extensive studies have explored solvent engineering, additive incorporation, dimensionality control, and interfacial modification in WBG perovskites, these approaches are often discussed independently [33, 34, 35, 36, 37]. However, the mechanistic connections among these strategies, especially in Br‐rich systems, have not been systematically examined from an intermediate‐chemistry perspective. Clarifying how intermediate lifetime, coordination symmetry, and phase persistence govern crystallization pathways is therefore essential for rational materials design.

This review presents an intermediate‐centric perspective on crystallization in Br‐enriched WBG perovskites. The chemical origin and classification of crystallization intermediates are examined from a coordination‐chemistry standpoint, encompassing both coordination‐defined species and structural intermediates such as polytypes. Halide‐segregated crystallization behavior is then analyzed as a composition‐dependent kinetic phenomenon. Building on this foundation, solvent‐mediated and additive‐engineered strategies that regulate intermediate formation, stabilization, synchronization, reconstruction, or elimination are systematically discussed. By framing crystallization control as deliberate programming of intermediate evolution, this review provides a coherent conceptual framework linking precursor chemistry to halide homogeneity, defect suppression, and operational stability in WBG perovskite photovoltaics.

## 2 Chemical Aspects of Crystallization in Br‐Rich Perovskites

### 2.1 Chemical Nature of Crystallization Intermediates

In solution‐processed halide perovskites, crystallization intermediates broadly encompass transient states that emerge during the transformation from precursor species to the final perovskite lattice. For the purpose of this review, these intermediates are broadly distinguished as chemical and structural intermediates according to their predominant characteristics. Chemical intermediates are characterized primarily by their transient composition and coordination environment, such as PbX_2_–solvent and AX–PbX_2_–solvent adducts [31]. In contrast, structural intermediates are characterized primarily by distinct lattice topology and octahedral connectivity, as exemplified by polytype‐related states such as 2H, 4H, 6H, and 8H observed during perovskite crystallization [29, 38]. These two types of intermediates do not necessarily constitute a universal sequential pathway; rather, their occurrence and coupling depend on composition and processing conditions. In this section, the chemical nature of crystallization intermediates is discussed primarily in terms of the coordination interactions that govern their formation and stability, while polytype‐related structural intermediates are discussed separately in Section 2.3.

From a chemical perspective, the stabilization of such intermediates is governed primarily by Lewis acid–base interactions involving the Pb^2+^ center and by hydrogen‐bonding interactions associated with the organic A‐site cation [39]. In the former case, Lewis‐basic solvent molecules or ligands coordinate to undercoordinated Pb^2+^ ions, partially substituting Pb–X coordination bonds and forming locally defined PbX_2_–solvent adduct–like states. In the latter case, solvents or additives with strong hydrogen‐bond‐donating or ‐accepting capability preferentially interact with the A‐site cation, yielding perovskite‐like frameworks in which solvent molecules are incorporated without extensive disruption of the BX_6_ octahedral connectivity. Importantly, these two coordination‐based stabilization modes are not merely different structural manifestations of transient species. Instead, they impose distinct kinetic constraints on nucleation, crystal growth, and lattice reorganization, thereby governing the accessibility of specific crystallization pathways and the lifetime of intermediate states during film formation.

This chemically grounded classification becomes particularly relevant for Br‐rich WBG perovskites. In Br‐rich compositions, the more rigid and strongly bound Pb–Br coordination network imposes a substantially higher energetic penalty for the local rearrangement of Pb─X bonds required to accommodate solvent coordination, thereby rendering the formation of PbX_2_–solvent adduct–type intermediate states less kinetically accessible under typical processing conditions. As a consequence, in many reported cases, the range of kinetically accessible intermediate configurations becomes significantly narrowed, and crystallization tends to proceed through pathways that do not rely on extensive disruption of the Pb–X framework. Under such conditions, Br‐rich perovskites often exhibit crystallization behavior in which different halide fractions follow distinct kinetic routes, rather than converging into a single, homogeneous pathway. In this context, identifying which type of intermediate state is thermodynamically accessible—and kinetically dominant—provides a direct conceptual link between precursor chemistry, halide‐segregated crystallization, and the emergence of microstructural and compositional heterogeneity in WBG perovskite films. Building on this chemical framework, the following subsection discusses how such intermediate‐controlled kinetics manifest experimentally as halide‐segregated crystallization pathways in Br‐rich perovskites.

### 2.2 Halide‐Segregated Crystallization Behavior

Mixed‐halide perovskites are representative WBG systems in which crystallization kinetics are strongly coupled to halide chemistry. In situ GIWAXS studies on MAPb(Br_*x*_I_1−*x*_)_3_ thin films (MA = methylammonium), comparing nitrogen gas quenching and the lead acetate route, demonstrate that halide segregation reflects an intrinsic characteristic of mixed‐halide crystallization rather than a route‐dependent effect (Figure 1a,b) [40]. Despite substantial differences in precursor chemistry and the nature of intermediate states, both fabrication routes exhibit a common crystallization motif.

**FIGURE 1:**
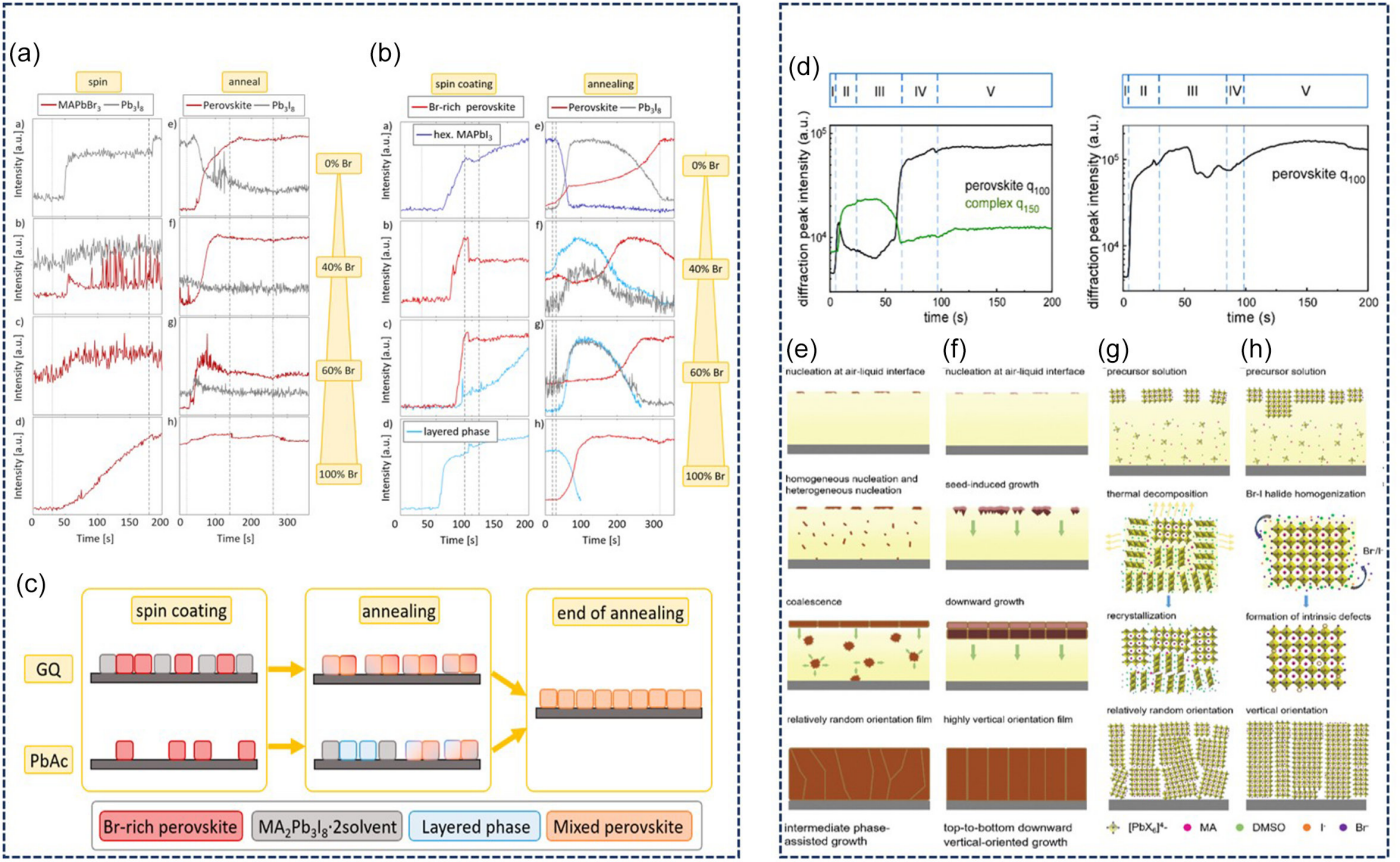
Halide‐segregated crystallization behavior in Br‐rich perovskites. (a–c) Route‐independent halide‐segregated crystallization. (a,b) Normalized integrated peak intensities extracted from radial profiles of GIWAXS reciprocal space maps as a function of time during spin coating and annealing for films prepared via (a) N_2_ gas quenching and (b) the lead acetate route, with bromide contents of 0% (MAPbI_3_), 40%, 60%, and 100%. Pb_3_I_8_ denotes the solvent‐coordinated MA_2_Pb_3_I_8_ intermediate (DMSO or DMF). (c) Schematic illustration of crystallization pathways in mixed‐halide perovskite thin films (GQ: gas quenching; PbAc: lead acetate route). (a–c) Reproduced with permission from [40], copyright 2024, American Chemical Society. (d–h) Br‐content‐dependent crystallization kinetics. (d) In situ GIWAXS measurements during thermal annealing of mixed‐halide perovskites, MAPbI_2_Br (left) and MAPbIBr_2_ (right), showing the time evolution of characteristic diffraction peak intensities. (e–h) Schematic illustrations of crystallization kinetics as a function of Br content: (e,g) intermediate phase–assisted growth in MAPbI_2_Br and (f,h) top‐to‐bottom downward growth in MAPbIBr_2_. (d–h) Reproduced with permission from [41], copyright 2025, Royal Society of Chemistry.

Time‐resolved GIWAXS measurements reveal that bromide‐ and iodide‐containing components follow distinct and parallel crystallization pathways. Bromide‐rich fractions crystallize rapidly and directly into the perovskite lattice during spin coating, whereas iodide‐rich components preferentially form intermediate states and convert into the perovskite structure only upon thermal annealing. Consequently, mixed‐halide films do not form via homogeneous cocrystallization but evolve through an initially halide‐segregated growth state (Figure 1c), in which a Br‐rich perovskite framework forms first and subsequently incorporates iodide during annealing‐driven intermixing. This process is accompanied by transient peak broadening in GIWAXS profiles, reflecting local compositional disorder prior to the formation of a macroscopically mixed film.

Notably, this segregated crystallization behavior persists irrespective of the deposition route, even though the specific intermediate states differ between solvent‐coordination‐dominated and layered low‐dimensional motifs. This robustness indicates that intermediates function as kinetic modulators rather than route‐specific byproducts, predefining crystallization pathways in mixed‐halide perovskites. Such early‐stage compositional inhomogeneities may persist into the final films and act as precursors for light‐induced halide segregation during device operation.

The composition dependence of this behavior is further illustrated in MAPbI_3−*x*_Br_*x*_ perovskites with moderate‐to‐high Br content (*x* = 1–2) [41]. For compositions such as MAPbI_2_Br with a bandgap (*E*_g_) of 1.77 eV, crystallization proceeds via a well‐defined solvent‐coordinated intermediate state, which transiently stabilizes Pb–halide frameworks and delays direct crystallization, thereby enabling halide redistribution and improved compositional homogeneity (Figure 1d, left). In contrast, Br‐rich compositions such as MAPbIBr_2_ (*E*_g_ = 1.95 eV) exhibit a suppression of detectable intermediate states, leading to rapid desolvation, direct perovskite formation, and downward crystallization initiated at the air–liquid interface (Figure 1d, right).

While this intermediate‐free pathway promotes preferential orientation and vertical charge transport, it also accelerates halide redistribution and defect formation through dissolution–recrystallization processes, resulting in reduced carrier lifetimes and enhanced nonradiative recombination. Overall, these observations demonstrate that the Br‐content‐dependent emergence or suppression of intermediate states critically governs halide redistribution, defect formation, and performance trade‐offs in mixed‐halide WBG perovskite absorbers (Figure 1e–h).

### 2.3 Polytype Intermediates in Halide‐Driven Crystallization Pathways

Intermediate phases typically refer to solvent‐coordinated complexes such as MAI–PbI_2_–DMSO, while polytypes (2H, 4H, and 6H) can be more broadly regarded as structural intermediates that emerge during crystallization through compositional reorganization. Polytype phases in mixed‐halide perovskites are frequently observed during film formation and are often discussed as competing crystalline phases; however, in the context of crystallization pathways, they are more appropriately understood as structural intermediates.

In mixed I/Br systems, the formation and evolution of polytypes are closely associated with halide composition, as illustrated in Figure 2a [38]. Qin et al. established a crystallization framework using a mixed‐halide perovskite composition (e.g., FA_0.83_MA_0.17_Pb(I_0.83_Br_0.17_)_3_), demonstrating that the film formation proceeds through a sequential evolution from the hexagonal 2H phase to higher‐order polytypes (4H and 6H), accompanied by the emergence of solvent‐coordinated intermediates and the perovskite α‐phase. In this framework, polytype phases are not isolated competing structures but arise as part of a continuous crystallization pathway. The incorporation of bromide is associated with a more rapid transition toward the perovskite phase, indicating its role in modulating crystallization kinetics. This behavior is often discussed in relation to halide‐dependent structural preferences, where iodide‐rich environments tend to stabilize face‐sharing octahedral configurations, while bromide incorporation facilitates the transition toward corner‐sharing perovskite frameworks. Notably, Figure 2a further shows that Cs incorporation significantly alters this pathway by suppressing the formation of higher‐order polytypes and promoting the direct formation of the perovskite α‐phase, effectively shortening the sequence of structural intermediates.

**FIGURE 2:**
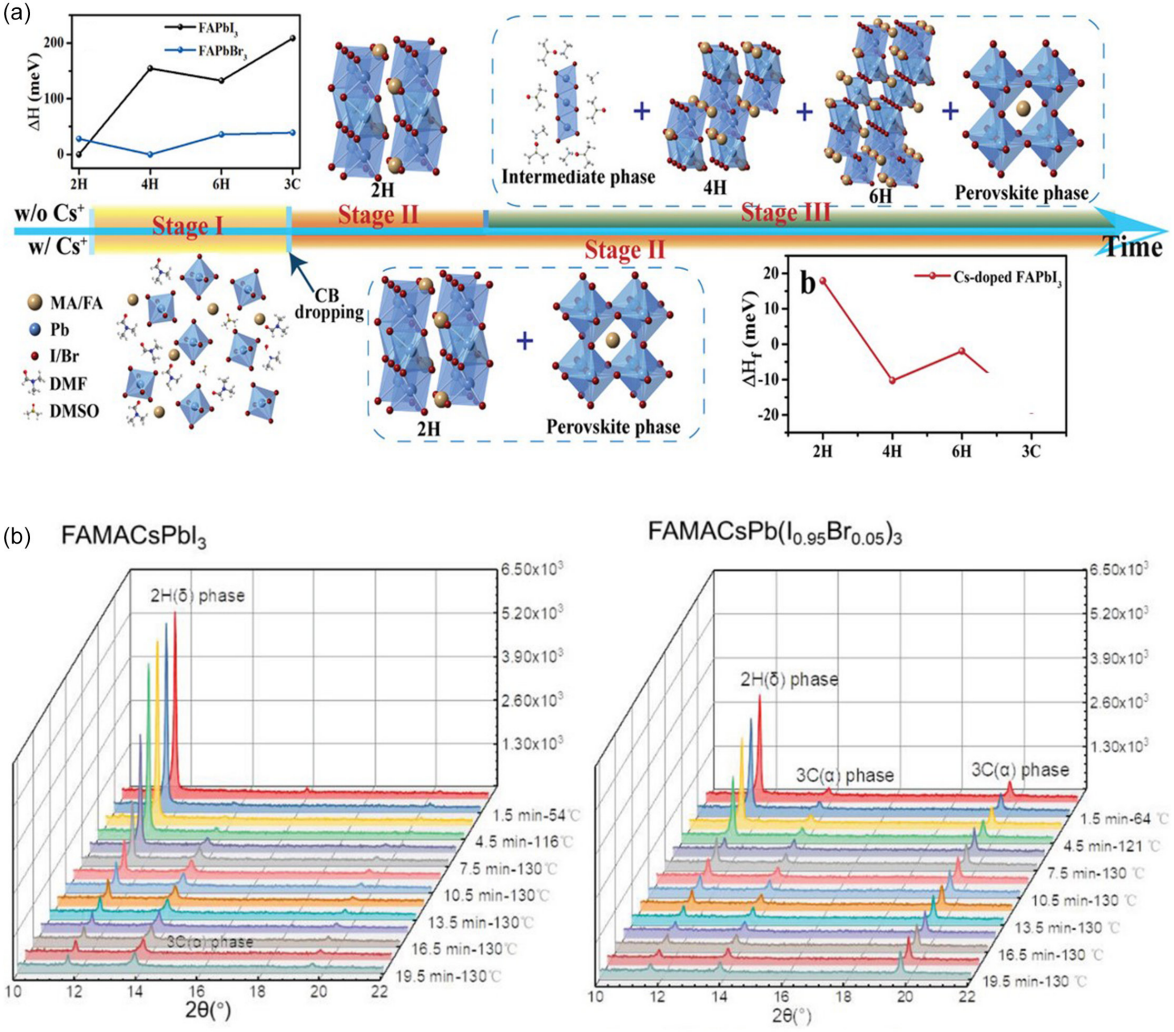
Polytype formation and phase transition pathways in mixed‐halide perovskites. (a) Schematic illustration of phase transition pathways during spin‐coating, comparing systems without and with Cs^+^ incorporation. Insets show DFT–calculated formation energies (Δ*H*) of FAPbI_3_ and FAPbBr_3_ and the formation energy gain at 12.5% Cs^+^ incorporation. “Intermediate phases” denote solvent‐coordinated species, whereas polytypes (2H, 4H, and 6H) represent structural intermediates. Reproduced with permission from [38], copyright 2019, Wiley‐VCH GmbH. (b) In situ XRD of FA_0.9_MA_0.05_Cs_0.05_PbI_3_ and FA_0.9_MA_0.05_Cs_0.05_Pb(I_0.95_Br_0.05_)_3_ perovskite films during annealing at 130 °C. Reproduced with permission from [29], copyright 2023, Wiley‐VCH GmbH.

This conceptual framework is further supported by complementary in situ experimental studies reported by He and coworkers [29]. In situ X‐ray diffraction (XRD) measurements (Figure 2b) for FA_0.9_MA_0.05_Cs_0.05_PbI_3_ and FA_0.9_MA_0.05_Cs_0.05_Pb(I_0.95_Br_0.05_)_3_ films reveal a sequential transformation from the 2H phase to higher‐order polytypes (4H, 6H, and 8H) and ultimately to the cubic perovskite (3C) phase, confirming the polytype‐mediated crystallization pathway. While Cs incorporation has been shown to shorten this pathway by suppressing intermediate polytypes, bromine incorporation primarily accelerates the phase transition kinetics along the same pathway. Specifically, the introduction of Br reduces the formation energy of the 3C phase and lowers the energy barriers associated with polytype transformations, thereby promoting faster conversion to the perovskite phase and enhancing overall crystallization kinetics.

Importantly, both studies indicate that polytype evolution is strongly influenced by halide composition, which modulates the relative stability of intermediate structures and the transition barriers along the crystallization pathway. Consistent with this, theoretical analyses suggest that halide incorporation alters both formation energies and kinetic barriers, highlighting its role in governing phase evolution. From a kinetic perspective, polytype intermediates exhibit pronounced temporal dependence: the 2H phase forms rapidly following supersaturation but remains metastable, while higher‐order polytypes emerge at later stages and may persist if not efficiently converted. This behavior defines a limited “annealing window” during which these intermediates can transform into the thermodynamically stable α‐phase. Furthermore, polytype formation is not independent of other intermediate pathways. The emergence of iodide‐rich polytypes can be coupled with excess iodide species that stabilize solvent‐coordinated complexes, illustrating that structural and chemical intermediates can be coupled during crystallization.

Overall, polytype formation in mixed‐halide perovskites should be regarded as a halide‐influenced structural intermediate pathway rather than merely competing phase formation. In this context, cation engineering (e.g., Cs incorporation) primarily modifies the crystallization pathway by suppressing intermediate states, whereas halide engineering (e.g., Br incorporation) predominantly influences the energetics and kinetics of phase transitions by altering formation energies and lowering transition barriers. Controlling both effects is therefore critical for achieving phase‐pure perovskite films and reproducible device performance.

## 3 Solvent‐Mediated Control of Crystallization Intermediates

### 3.1 Competing Halide Complexes and Solvent–Adduct Intermediates

In Br‐rich WBG perovskites, crystallization pathways are highly sensitive to the balance between halide‐dominated prenucleation species and solvent‐stabilized intermediate states, owing to the intrinsically strong Pb–X interactions that favor rapid and poorly controlled nucleation. This competition is well illustrated by a recent study employing a green solvent system to deliberately reshape the intermediate landscape in Br‐rich WBG compositions (Figure 3a) [42].

**FIGURE 3:**
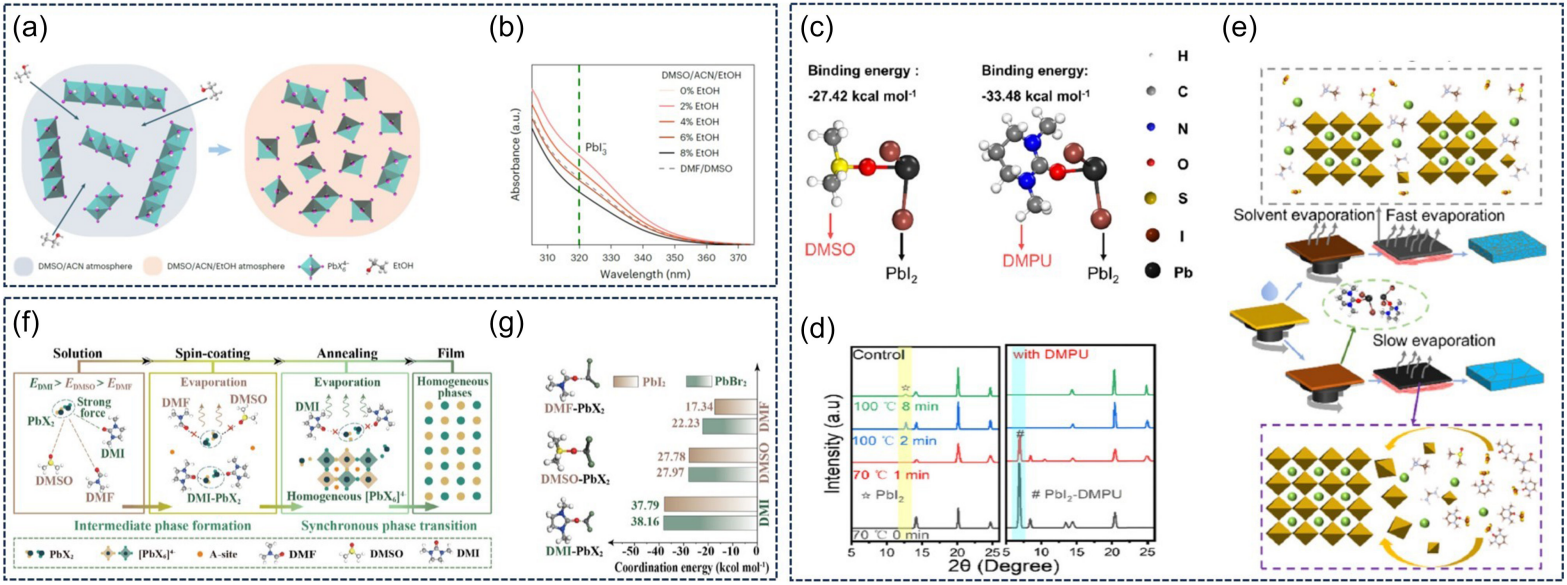
Solvent‐mediated control of crystallization intermediates in Br‐rich perovskites. (a, b) EtOH‐induced reconstruction of crystallization intermediates in DMSO/ACN‐based precursor solutions. (a) Schematic illustration of solvent–PbX_2_ intermediate reconstruction induced by EtOH addition. (b) UV–vis absorption spectra of precursor solutions with increasing EtOH content. (a, b) Reproduced with permission from [42], copyright 2024, Springer Nature. (c–e) Stabilization of solvent–adduct intermediates enabled by introducing DMPU into DMSO‐based solvent systems. (c) Comparison of PbI_2_–solvent binding energies for DMSO and DMPU. (d) XRD patterns of perovskite films without and with DMPU at different annealing temperatures and times. (e) Schematic illustration comparing crystallization pathways without DMPU (top) and with DMPU (bottom). (c–e) Reproduced with permission from [43], copyright 2024, Royal Society of Chemistry. (f, g) Synchronization of halide crystallization via halide‐symmetric solvent coordination using DMI. (f) Schematic illustration of homogeneous halogen‐phase perovskite film formation via DMI–PbX_2_ adducted intermediates. (g) Coordination energies of PbX_2_ with different solvents. (f, g) Reproduced with permission from [44], copyright 2025, Wiley‐VCH GmbH.

The representative composition FA_0.65_Cs_0.35_PbI_1.8_Br_1.2_ (FA = formamidinium; Br fraction = 40%, *E*_g_ = 1.78 eV) exemplifies a Br‐rich system in which solution chemistry critically dictates crystallization behavior. In dimethyl sulfoxide (DMSO)/acetonitrile (ACN) solvent mixtures without protic additives, the low coordinating ability of acetonitrile favors direct halide coordination to Pb^2+^, leading to the formation of halide‐rich Pb–X complexes (for example, PbI_3_^−^‐like species). These halide‐dominated species readily aggregate into colloidal clusters, triggering rapid, spatially heterogeneous nucleation during solvent evaporation and resulting in a narrow processing window and poor film uniformity. In this regime, crystallization is effectively governed by halide‐driven prenucleation chemistry rather than by well‐defined solvent‐stabilized intermediate states.

The introduction of a small amount of ethanol (EtOH) fundamentally reconstructs this chemical landscape, not by directly coordinating to Pb^2+^, but by preferentially interacting with halide anions. Owing to its high anion‐accepting ability, EtOH partially sequesters I^−^ and Br^−^ ions, thereby suppressing their direct coordination to Pb^2+^ and inhibiting the formation of halide‐rich Pb–X complexes. As a consequence, the population of halide‐dominated prenucleation species is markedly reduced, and a larger fraction of Pb^2+^ becomes available for coordination with DMSO, promoting the formation of solvent–PbX_2_ adduct–like intermediate states.

This EtOH‐induced shift in coordination equilibrium is directly evidenced by the reduction of spectral signatures associated with halide‐rich Pb–X complexes and the concomitant stabilization of DMSO–PbX_2_ adduct states, as confirmed by UV–vis absorption, Fourier‐transform infrared (FTIR) spectroscopy, and nuclear magnetic resonance (NMR) analyses (Figure 3b). Importantly, these solvent–PbX_2_ adduct–like states do not immediately convert into the perovskite lattice upon partial solvent removal. Instead, they act as metastable intermediate states that delay nucleation and enable a more homogeneous spatial distribution of nuclei during film deposition.

As a result, EtOH‐containing inks yield compact, void‐free films with well‐controlled grain morphology, which is particularly beneficial for Br‐rich WBG perovskites where excessive halide activity would otherwise drive uncontrolled crystallization. The same intermediate‐control mechanism is also operative in compositions with lower but still substantial Br content, such as Cs_0.05_FA_0.80_MA_0.15_PbI_2.25_Br_0.75_ (Br fraction = 25%, *E*_g_ = 1.68 eV), underscoring the generality of solvent–PbX_2_ intermediate engineering across Br‐rich WBG systems.

Overall, this study highlights that, in Br‐rich perovskites, crystallization control is not achieved merely by changing the solvent environment but by deliberately steering the competition between halide‐dominated prenucleation complexes and solvent‐stabilized PbX_2_ adduct–like intermediate states. Manipulating this balance emerges as a central design principle for achieving regulated nucleation, uniform film formation, and robust processing windows in WBG‐perovskite photovoltaics.

### 3.2 Stabilizing Solvent–Adduct Intermediates via Enhanced Solvent Coordination

Beyond modulating the formation of crystallization intermediates, an effective strategy for WBG perovskites is to stabilize solvent–adduct intermediates through enhanced solvent coordination, thereby extending their lifetime and decoupling intermediate evolution from abrupt crystallization. In this framework, solvent–precursor interaction strength determines not only the emergence of intermediates but also their persistence during solvent removal and thermal annealing, ultimately defining crystallization kinetics and the accessible processing window.

This concept is illustrated in a Br‐containing WBG perovskite, FA_0.8_Cs_0.15_MA_0.05_PbI_2.4_Br_0.6_ (Br fraction = 20%, *E*_g_ = 1.65 eV), which exhibits rapid crystallization and a narrow antisolvent window in conventional solvent systems [43]. In *N*,*N‐*dimethylformamide (DMF)/DMSO‐based inks, solvent evaporation, intermediate collapse, and perovskite crystallization occur in close succession, limiting kinetic control. Partial substitution of DMSO with 1,3‐dimethyl‐3,4,5,6‐tetrahydro‐2(1H)‐pyrimidinone (DMPU) strengthens solvent–PbX_2_ coordination, leading to the formation of more stable solvent‐coordinated intermediates, as supported by increased solvent–PbI_2_ binding energies (Figure 3c).

As a consequence, these intermediates persist into the annealing stage and undergo gradual rather than abrupt conversion to the perovskite phase, as evidenced by temperature‐ and time‐dependent XRD measurements (Figure 3d). This prolonged intermediate lifetime effectively decouples antisolvent timing from crystallization outcomes, broadening the processing window and reducing sensitivity to processing fluctuations (Figure 3e).

Beyond strongly coordinating additives such as DMPU, N‐methyl‐2‐pyrrolidone (NMP) provides a representative case in which solvent–PbX_2_–derived intermediates remain persistent under scalable deposition conditions [45]. In a MA‐free Br‐rich WBG perovskite, FA_0.9_Cs_0.1_Pb(I_0.6_Br_0.4_)_3_ (*E*_g_ = 1.8 eV), NMP forms a solvent‐coordinated intermediate, identified as an FAI·PbI_2_·NMP adduct, that survives solvent extraction and requires thermal activation for conversion to the perovskite phase. Compared with DMF/DMSO systems, the low vapor pressure and coordinative interaction of NMP suppress premature solvent loss and delay crystallization until annealing.

Although intermediate stabilization in the NMP system is likewise governed by solvent–PbX_2_ coordination, its significance lies in demonstrating that extended intermediate persistence can be exploited to regulate crystallization under scalable processing conditions, such as blade coating, where prolonged drying would otherwise induce uncontrolled nucleation.

Overall, these studies establish stabilization of solvent–adduct intermediates via enhanced solvent coordination as a general design principle for WBG perovskites. For Br‐containing compositions with intrinsically fast crystallization kinetics, prolonging intermediate lifetime—through either strengthened coordination or suppressed solvent volatilization—provides an effective route to reproducible film formation, widened processing windows, and reduced defect formation.

### 3.3 Synchronizing Solvent–Adduct Intermediates via Halide‐Symmetric Solvent Coordination

A representative example of solvent‐mediated regulation of crystallization intermediates in Br‐rich WBG perovskites is reported by Li et al., who investigated a mixed‐halide perovskite with the composition FA_0.78_Cs_0.15_DMA_0.07_Pb(I_0.7_Br_0.3_)_3_ (*E*_g_ = 1.76 eV) [44]. At this bromide concentration, the perovskite system is intrinsically prone to asynchronous crystallization, in which Br‐rich domains nucleate and crystallize more rapidly than I‐rich components, leading to pronounced halide inhomogeneity.

To address this challenge, the authors employed a mixed‐solvent system in which DMSO was partially replaced by 1,3‐dimethyl‐2‐imidazolidinone (DMI) (DMF:DMSO:DMI = 4:0.6:0.4). Rather than using DMI as a single solvent, this approach exploits the differential evaporation behavior of the solvent components during spin‐coating. As DMF and DMSO evaporate rapidly, the high‐boiling‐point DMI remains in the wet film and selectively coordinates with lead halide species, resulting in the formation of a DMI‐dominated solvent–adduct intermediate state. The overall concept of homogeneous intermediate formation and its role in regulating subsequent crystallization are schematically illustrated in Figure 3f.

DFT calculations reveal that DMI exhibits strong and nearly identical coordination energies toward PbI_2_ and PbBr_2_, in contrast to conventional coordinating solvents such as DMF and DMSO (Figure 3g). This halide–symmetric interaction suppresses the preferential stabilization of either halide component and enables the formation of a chemically homogeneous intermediate state. Spectroscopic analyses, including FTIR and liquid‐state ^1^H NMR, further confirm strong Pb–O coordination between DMI and lead halide species, while XRD and in situ GIWAXS measurements identify a distinct DMI‐derived solvent–adduct intermediate phase in the as‐deposited wet films.

Crucially, this intermediate state remains stable during the spin‐coating stage and only undergoes decomposition during thermal annealing when gradual DMI desorption occurs. In situ GIWAXS and optical spectroscopy demonstrate that this delayed conversion enforces synchronous crystallization of iodide‐ and bromide‐containing frameworks, in sharp contrast to the sequential crystallization observed in conventional solvent systems. As a result, the final perovskite films exhibit a uniform I/Br distribution, reduced lattice strain, and suppressed halide segregation.

Overall, this work highlights a key design principle for solvent‐mediated intermediate control in Br‐rich WBG perovskites: effective synchronization of halide crystallization requires not only strong solvent–precursor coordination but also halide‐symmetric binding energetics combined with appropriately tuned solvent volatilization kinetics. In this context, solvent‐derived intermediate states function as active regulators of crystallization pathways rather than passive crystallization retardants.

### 3.4 Antisolvent‐Mediated Control of Intermediate Evolution

Beyond precursor–solvent coordination, antisolvent treatment provides an additional kinetic lever for regulating intermediate evolution during film formation. This role is particularly important in mixed‐halide WBG perovskites, where differences in I^−^ and Br^−^ coordination and crystallization kinetics can generate compositionally distinct transient phases before formation of the final mixed‐halide lattice. As discussed above, antisolvent‐triggered crystallization pathways can vary strongly with Br content: MAPbI_2_Br crystallizes through a solvent–complex intermediate, whereas the more Br‐rich MAPbIBr_2_ composition undergoes direct conversion to the cubic perovskite without a detectable intermediate [41]. Antisolvent processing therefore operates within a composition‐dependent crystallization landscape in which the accessibility and evolution of intermediate states are increasingly constrained as the Br fraction changes.

A direct example of antisolvent‐mediated intermediate control was demonstrated for the mixed‐halide WBG composition FA_0_._83_Cs_0_._17_Pb(I_0_._8_Br_0_._2_)_3_ (*E*_g_ = 1.67 eV) using ethyl acetate (EA) mixed with isopropanol (IPA) [46]. With EA alone, the unannealed film contained PbI_2_·NMP intermediates with both (011) and (020) orientations, together with PbI_2_·DMF_0_._5_·NMP_0_._5_, δ‐phase, and residual PbI_2_. The corresponding PL spectrum also exhibited a strongly blueshifted feature assigned to a transient Br‐rich perovskite phase, followed by substantial spectral evolution during annealing toward the final mixed‐halide composition. Upon addition of IPA to EA, the dominant orientation of the PbI_2_·NMP intermediate shifted from (011) toward (020), while the δ‐phase and PbI_2_ were no longer detected in the unannealed films, and α‐phase formation was enhanced. Concurrently, the initial PL approached that of the final composition and underwent much smaller shifts during subsequent annealing, indicating suppressed halide redistribution. Excessive IPA, however, partially disrupted the PbI_2_·NMP adduct and resulted in residual PbI_2_ after annealing. These observations show that, in WBG mixed‐halide perovskites, antisolvent composition can regulate the phase assemblage, orientation, and conversion of solvent‐coordinated intermediates while simultaneously influencing early‐stage halide redistribution. Appropriate control of solvent extraction and precursor–solvent coordination is therefore important for avoiding compositionally heterogeneous crystallization pathways and promoting homogeneous WBG perovskite formation.

### 3.5 Design Principles for Solvent‐Mediated Intermediate Control

Solvent‐mediated control of crystallization intermediates provides a chemically grounded framework for regulating nucleation and growth in Br‐rich WBG perovskites, where strong Pb–X interactions otherwise favor rapid and poorly controlled crystallization. As demonstrated by the case studies discussed above, solvents actively shape intermediate chemistry and kinetics rather than serving merely as dissolution media.

Across these studies, several interrelated design principles emerge. First, steering the competition between halide‐dominated prenucleation complexes and solvent–PbX_2_ adducts governs the onset and spatial uniformity of nucleation. Second, enhancing solvent–precursor coordination strength or suppressing solvent volatilization prolongs the lifetime of solvent‐derived intermediates, thereby decoupling solvent removal from abrupt crystallization. Third, employing solvents with halide‐symmetric coordination energetics synchronizes iodide‐ and bromide‐containing frameworks, mitigating halide‐segregated crystallization. In addition, antisolvent treatment provides a complementary kinetic leverage by regulating solvent extraction and the subsequent evolution of solvent‐coordinated intermediates.

Importantly, these strategies function not as isolated mechanisms but as complementary control knobs that can be rationally combined. By jointly tuning coordination strength, halide selectivity, and solvent removal kinetics, solvent‐derived intermediates can be deliberately programmed to regulate crystallization pathways, processing windows, and film uniformity. This intermediate‐centric perspective provides a coherent design framework for translating solvent chemistry into reproducible processing strategies for high‐performance Br‐rich WBG‐perovskite photovoltaics.

## 4 Additive‐Engineered Control of Crystallization Intermediates

### 4.1 MACl‐Regulated Crystallization Intermediates

Additive engineering in Br‐rich WBG perovskites is increasingly understood not merely as defect passivation or compositional tuning but as a strategy to reconfigure the Pb^2+^–X^−^–solvent coordination environment that governs crystallization intermediates. Among various additives, methylammonium chloride (MACl) has emerged as a representative case whose impact is highly sensitive to halide composition and solvent chemistry, giving rise to distinct—and sometimes opposing—crystallization pathways under different processing conditions. Rather than reflecting disparate mechanisms, recent studies indicate that these behaviors can be understood within a unified framework in which MACl modulates Pb–halide–solvent coordination and thereby alters the energetic accessibility of intermediate phases. Because of the strong Pb–Cl interaction, MACl competes with both Pb–Br/Pb–I bonding and Pb–solvent coordination, determining whether intermediate phases are stabilized, transformed, or bypassed altogether.

A representative example of intermediate stabilization–driven crystallization was reported by Shen et al., who introduced MACl into Br‐rich WBG perovskites with the composition FA_0.83_Cs_0.17_Pb(I_0.6_Br_0.4_)_3_ (*E*_g_ = 1.8 eV) [47]. In this DMF/DMSO cosolvent system, MACl promotes the formation of chloride‐rich Pb–halide–solvent intermediates at the earliest stages of crystallization, as revealed by distinct structural signatures during thermal annealing (Figure 4a,b). In situ XRD and GIWAXS measurements show reduced lattice parameters in the initial nuclei, consistent with transient Cl^−^ incorporation driven by stronger Pb–Cl coordination. These chloride‐rich intermediates subsequently evolve through hexagonal polytypes (4H and 6H), which act as metastable reservoirs supplying Br^−^ and I^−^ while Cl^−^ is gradually expelled during high‐temperature annealing (~150 °C). Time‐resolved PL measurements (Figure 4c,d) further indicate that this intermediate‐mediated pathway suppresses the formation of iodine‐rich, low‐bandgap emissive phases typically observed in Br‐rich systems. This controlled intermediate evolution ultimately leads to improved halide homogeneity, mitigated light‐induced halide segregation, and enhanced device performance.

**FIGURE 4:**
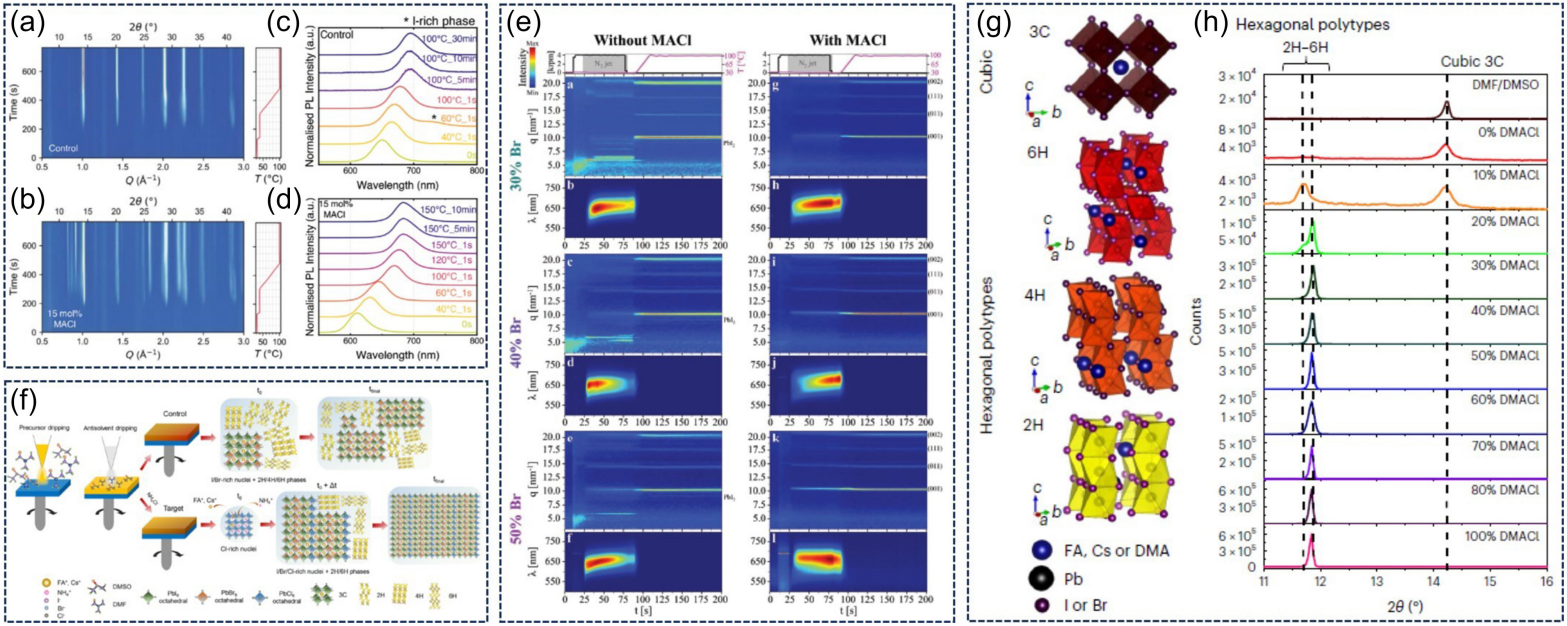
Chloride‐containing additive control of crystallization intermediates in Br‐rich perovskites. (a–d) Crystallization behavior of FA_0.83_Cs_0.17_Pb(I_0.6_Br_0.4_)_3_ thin films processed from a DMF/DMSO cosolvent system with MACl. (a,b) Time‐resolved GIWAXS patterns of films (a) without and (b) with MACl, recorded during thermal annealing. (c,d) Normalized PL spectra obtained after various thermal treatments for films (c) without and (d) with MACl. (a–d) Reproduced with permission from [47], copyright 2023, Wiley‐VCH GmbH. (e) Effect of Br content and MACl addition on the formation of FA_0.95_Cs_0.05_Pb(Br_*x*_I_1−*x*_)_3_ (0.3 ≤ *x* ≤ 0.5) perovskite films processed from a DMF/NMP cosolvent system, revealed by in situ GIWAXS and PL intensity maps during spin coating and thermal annealing. Reproduced with permission from [48], copyright 2024, Wiley‐VCH GmbH. (f) Schematic illustration of NH4Cl‐mediated crystallization modulation in wide‐bandgap perovskite films. Reproduced with permission from [49], copyright 2025, Wiley‐VCH GmbH. (g,h) Impact of DMACl on the crystallization intermediates of FA_0.83_Cs_0.17_Pb(Br_0.2_I_0.8_)_3_. (g) Unit‐cell structures of hexagonal perovskite polytypes (2H, 4H, and 6H) illustrating face‐sharing PbI_6_ octahedral connectivity, compared with the corner‐sharing cubic (3C) perovskite phase. (h) X‐ray diffraction patterns of thin films processed with increasing DMACl content, revealing the stabilization of hexagonal polytype intermediates and their evolution toward the cubic perovskite phase. (g,h) Reproduced with permission from [50], copyright 2022, Springer Nature.

An opposite yet mechanistically consistent behavior—intermediate suppression–driven crystallization—was demonstrated by Guaita et al. in Cs_0.05_FA_0.95_Pb(Br_*x*_I_1−*x*_)_3_ (0.3 ≤ *x* ≤ 0.5), with *E*_g_ = 1.68–1.86 eV [48]. Using DMF/NMP cosolvents combined with N_2_ gas quenching, the authors showed that, in the absence of MACl, Br‐rich films form stable solvent‐coordinated crystalline intermediates, such as FAPb(Br,I)_3_·(DMF,NMP) and Pb(Br,I)_2_·NMP, which compete with perovskite lattice growth, particularly at intermediate Br contents (30%–40%). Upon MACl addition, these solvent‐coordinated intermediates are no longer detected by in situ GIWAXS; instead, crystallization proceeds directly through a perovskite nanocrystalline phase during spin‐coating, as evidenced by composition‐ and time‐resolved GIWAXS and PL intensity maps (Figure 4e). In this NMP‐based system, strong Pb–Cl interactions effectively weaken Pb–solvent coordination, preventing intermediate stabilization and favoring rapid perovskite nucleation. Chloride participates only transiently at early stages and fully volatilizes during annealing, leaving the final halide stoichiometry unchanged. The suppression of intermediate trapping leads to enhanced crystallinity and improved Br/I homogeneity, as confirmed by nano–X‐ray fluorescence (XRF) mapping.

Taken together, these studies demonstrate that MACl regulates crystallization not by imposing a specific intermediate phase, but by modulating the Pb–halide–solvent coordination landscape that defines intermediate accessibility. Depending on solvent donicity and halide composition, this modulation manifests either as stabilization of chloride‐rich intermediates that mediate phase‐selective conversion or as suppression of solvent‐coordinated intermediates that enable direct perovskite nucleation. This coordination‐driven perspective reconciles previously conflicting observations and underscores crystallization intermediates as an emergent consequence of precursor chemistry rather than an intrinsic function of the additive itself.

### 4.2 A‐Site–Participating Chloride–Mediated Crystallization Intermediates

Crystallization control in Br‐rich WBG mixed‐halide perovskites can be further advanced by employing ammonium‐based chloride additives that engage directly in cation‐assisted intermediate evolution. In contrast to MACl, where MA^+^ primarily modulates halide coordination and nucleation kinetics without forming a resolvable crystalline intermediate phase, both NH_4_Cl and DMACl introduce cation‐involved intermediate pathways that more directly reshape phase evolution during film formation. Importantly, these two additives represent distinct levels of intermediate involvement, spanning from highly transient ammonium‐assisted nuclei to structurally resolvable polytype intermediates.

In Br‐rich systems such as FA_0.8_Cs_0.2_Pb(I_0.7_Br_0.3_), corresponding to a bandgap of approximately 1.7–1.75 eV, crystallization is strongly dictated by the intermediate phase landscape, including solvent‐coordinated adducts and hexagonal polytypes. In the absence of ammonium chloride additives, strong Pb^2+^–solvent coordination in DMF/DMSO stabilizes these intermediates and allows hexagonal polytypes (2H/4H/6H) to coexist with cubic nuclei, thereby impeding phase‐pure cubic growth and leading to stacking disorder, microstrain, and incomplete halide homogenization [49]. The introduction of NH_4_Cl alters this intermediate landscape by weakening precursor–solvent coordination and suppressing long‐lived adduct species. Crystallization instead proceeds through Cl‐rich NH_4_^+^‐associated transient intermediates that promote early nucleation of the cubic perovskite framework, as schematically illustrated in Figure 4f. The NH_4_^+^ cation functions as a transient A‐site species temporarily associated with early nuclei and is subsequently removed as NH_3_ during annealing, thereby moderating nucleation kinetics and enabling gradual Br^−^/I^−^ redistribution without residual ammonium incorporation in the final lattice.

A more explicit and structurally defined mode of ammonium‐mediated intermediate engineering is realized with DMACl [50]. Owing to the steric and symmetry mismatch of DMA^+^ with the cubic perovskite lattice, crystallization proceeds via well‐resolved hexagonal face‐sharing polytype intermediates (2H/4H/6H) that incorporate DMA^+^ into their framework. The crystallographic nature of these intermediates, including the face‐sharing connectivity of PbI_6_ octahedra in contrast to the corner‐sharing cubic (3C) phase, is illustrated in Figure 4g. Consistent with this structural picture, Figure 4h shows XRD patterns in which these DMA‐containing polytypes are clearly stabilized during early‐stage annealing. Unlike the highly transient NH_4_^+^‐associated species, these intermediates persist on a minute‐scale at low temperature before being thermally expelled, enabling controlled conversion into the final 3C cubic perovskite phase with reduced microstrain, strong crystallographic texture, and enhanced halide homogeneity, even at Br contents of 20%–40%.

Taken together, NH_4_Cl and DMACl illustrate a continuum of ammonium–chloride–mediated crystallization control, spanning from transient, chemically driven intermediate modulation to structurally resolvable intermediate‐phase evolution. This comparison highlights that the effectiveness of chloride additives in Br‐rich WBG perovskites is governed not merely by halide chemistry, but by the extent to which the accompanying A‐site cation interacts with—and subsequently withdraws from—the evolving intermediate phases. In this context, crystallization stability arises from controlled regulation of intermediate lifetime and structural persistence, rather than from simple halide modification.

### 4.3 Passively Modulated Crystallization Intermediates via A‐Site Cations

In Br‐rich mixed‐halide perovskites, compositional instability often originates from kinetically frozen halide heterogeneity established during intermediate evolution. Rather than generating distinct crystalline intermediates, certain A‐site additives operate by passively modulating the intermediate regime through kinetic regulation of nucleation and halide exchange.

A representative example is propane‐1,3‐diammonium iodide (PDA), applied to Br‐rich compositions such as Cs_0.15_FA_0.85_Pb(I_0.4_Br_0.6_)_3_ (*E*_g_ = 1.97 eV) [51]. During solution processing, the control system forms a heterogeneous ensemble of Br^−^ and I‐rich precursor fragments and nanocrystalline intermediates that rapidly lock into compositionally biased nuclei. Time‐resolved absorption mapping (Figure 5a,b) shows that PDA delays the onset of lattice formation and promotes more gradual spectral evolution, indicating an extended intermediate window prior to nucleation. This prolonged regime enables more complete Br^−^/I^−^ redistribution before lattice lock‐in. As illustrated in Figure 5c, PDA acts as a passive kinetic modulator that transiently mediates interactions among halide‐containing precursor species without forming a resolvable intermediate phase or contributing to the final perovskite framework. Consequently, depth‐dependent halide nonuniformity—particularly Br‐rich accumulation at buried interfaces—is significantly suppressed, leading to reduced defect density and improved operational stability.

**FIGURE 5:**
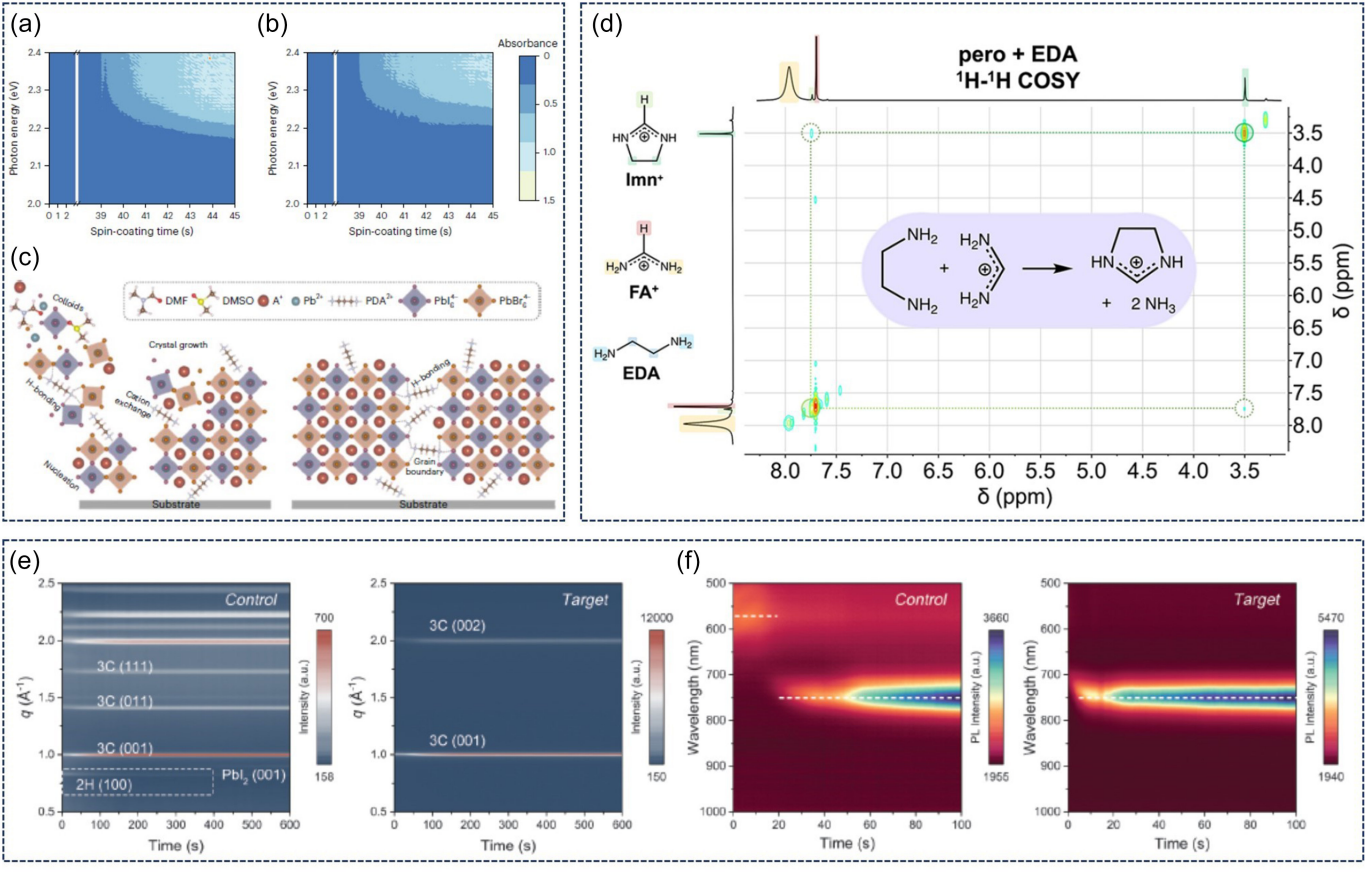
Organic cation–driven modulation of crystallization intermediates in Br‐rich perovskites. (a–c) Passively modulated crystallization intermediates induced by the PDA additive. (a,b) Time‐resolved absorption intensity maps recorded during spin coating of perovskite precursor films (a) without and (b) with 1 mol% PDA. Anisole antisolvent was dripped at ~39 s. (c) Schematic illustration of the interaction between PDA cations and mixed Br/I perovskite precursor species during film formation. Reproduced with permission from [51], copyright 2023, Springer Nature. (d) Actively reconstructed crystallization intermediates induced by the EDA additive. ^1^H–^1^H correlation spectroscopy (COSY) of (FA_0.83_Cs_0.17_)Pb(I_0.75_Br_0.25_)_3_ with 10 mol% EDA in d_6_‐DMSO. The inset depicts the reaction of FA^+^ with EDA to form Imn^+^ and ammonia. Reproduced with permission from [52], copyright 2022, ACS Publications. (e,f) Intermediate‐eliminating crystallization enabled by the OAmI additive. (e) False‐color maps of diffraction intensity as a function of scattering vector q and time from in situ GIWAXS measurements comparing control and OAmI‐treated target films. (f) Time‐resolved PL intensity maps as a function of emission wavelength for control and OAmI‐treated target films. Reproduced with permission from [53], copyright 2024, American Association for the Advancement of Science.

A conceptually related yet mechanistically distinct case is provided by imidazolium (IA) additives in Br‐rich perovskites such as Cs_0.2_FA_0.8_Pb(I_0.6_Br_0.4_)_3_ [54]. Unlike PDA, IA does not extend the intermediate lifetime through steric mediation but instead redistributes nucleation kinetics between Br^−^ and I‐rich species. Dynamic light scattering and in situ PL reveal that IA balances the formation rates of halide‐rich precursor clusters, suppressing preferential Br‐dominant nucleation. DFT further indicates preferential stabilization of solvent–PbBr_2_ adducts, mitigating the intrinsic thermodynamic bias toward Br‐rich nuclei. As a result, IA‐containing films exhibit smoother spectral evolution during crystallization and improved compositional homogeneity.

Importantly, both PDA and IA function as transient kinetic regulators during film formation. The resulting reductions in strain, defect density, and nonradiative recombination originate from intermediate‐stage modulation during crystallization. Collectively, these findings define a passive intermediate‐engineering regime in which compositional and structural homogenization are achieved through temporal regulation of nucleation and halide exchange without the formation of distinct, long‐lived intermediate phases.

### 4.4 Actively Reconstructed Crystallization Intermediates via Diammonium A‐Site Cations

In contrast to passive intermediate modulation, Br‐rich mixed‐halide perovskites can undergo active reconstruction of their crystallization intermediates upon incorporation of diammonium A‐site cations. These Br‐rich systems intrinsically stabilize long‐lived precursor‐ and polytype‐related intermediates, which serve as structural templates for local halide inhomogeneity and subsequently promote photoinduced phase segregation.

Diammonium cations address this instability by chemically restructuring the intermediate phase rather than merely tuning crystallization kinetics. A representative example is ethylenediamine (EDA), which drives a transformation of the precursor chemistry prior to lattice formation [52]. Solution‐phase nuclear magnetic resonance (NMR) analyses (Figure 5d) show that EDA reacts with FA^+^ to generate imidazolinium (Imn^+^) species before crystallization, thereby modifying precursor speciation and Pb–halide coordination environments. In situ GIWAXS measurements further reveal suppression of hexagonal polytype intermediates, including the 2H and 4H phases commonly observed in Br‐rich systems. Rather than eliminating intermediates entirely, EDA redirects crystallization through chemically transformed intermediate states that favor more homogeneous halide incorporation.

Consequently, PL mapping and compositional analyses confirm reduced halide segregation and improved compositional stability under illumination, even in high‐Br WBG absorbers. Collectively, these findings establish diammonium cation–induced intermediate reconstruction as an active crystallization‐control strategy, distinct from the passive kinetic modulation described in Section 4.3 and from dimensionality‐control approaches based on spacer cations. By redefining precursor chemistry prior to lattice consolidation, this approach mitigates the halide inhomogeneity that typically originates from long‐lived Br‐rich intermediates in WBG compositions.

### 4.5 Intermediate‐Eliminating Crystallization Pathways via Bulky Organic Ammonium Cations

Most crystallization strategies discussed above aim to stabilize, suppress, or reconstruct intermediate phases in Br‐rich WBG perovskites. Film formation in moderate‐Br WBG compositions is typically mediated by solvent‐coordinated adducts, Br‐rich precursor clusters, and transient hexagonal polytype nuclei, which predefine halide heterogeneity and structural disorder. In contrast, a nucleus‐first pathway enables crystallization that precludes the formation of such solvent‐ or halide‐segregated intermediates altogether.

The introduction of a small amount of bulky oleylammonium iodide (OAmI) redirects crystallization toward the immediate formation of halide‐homogeneous pseudocubic (3C) nuclei, effectively bypassing intermediate‐dominated growth [53]. This transition is directly visualized in Figure 5e,f. In situ GIWAXS false‐color maps (Figure 5e) reveal that control films exhibit transient Br‐rich and polytype‐related diffraction features during early crystallization, whereas OAmI‐treated films show immediate dominance of the 3C phase without detectable intermediate signatures. Consistently, time‐resolved PL intensity maps (Figure 5f) display early Br‐rich emission in control films but a single, continuously evolving emission in OAmI‐treated films, indicative of compositionally uniform nuclei. From an intermediate‐centric perspective, this behavior represents a distinct crystallization paradigm: rather than regulating intermediate evolution, the additive enforces early formation of homogeneous nuclei and thereby suppresses intermediate emergence. The resulting films exhibit reduced energetic disorder and diminished halide segregation, leading to enhanced quasi–Fermi‐level splitting and improved resistance to thermal stress, bias‐induced ion migration, and light‐induced phase segregation.

Overall, for moderate‐Br WBG perovskites, controlling which nuclei form before solvent‐ or halide‐driven intermediates can emerge may be more decisive than conventional intermediate engineering. This intermediate‐eliminating pathway thus complements passive modulation and active reconstruction strategies for stabilizing mixed‐halide perovskites.

### 4.6 Design Principles for Additive‐Engineered Intermediate Control

The studies discussed in Sections 4.1–4.5 reveal that crystallization intermediates in Br‐rich WBG mixed‐halide perovskites are not intrinsic phases, but emergent states governed by the Pb^2+^–halide–solvent coordination landscape. Additives shift this coordination balance, thereby regulating the formation and lifetime of intermediate phases. Strong Pb–Cl interactions may stabilize chloride‐rich intermediates or suppress solvent‐coordinated adducts, depending on solvent donicity and halide composition. In contrast, bulky or diammonium cations can alter precursor speciation and even reshape intermediate phase evolution. Additive engineering should therefore be understood as a means of programming crystallization pathways through coordination control rather than as conventional postcrystallization defect engineering.

Mechanistically, additive‐mediated control spans a continuum of intervention. Passive kinetic regulators extend the intermediate window without forming distinct structural phases, enabling improved halide redistribution before lattice lock‐in. Reconstruction strategies chemically redefine precursor species or stabilize polytype intermediates to redirect phase conversion. In contrast, intermediate‐eliminating approaches enforce early formation of homogeneous pseudocubic nuclei, bypassing solvent‐ and halide‐segregated intermediates. The effectiveness of each regime depends critically on solvent coordination strength, halide thermodynamics, and bromide fraction.

Thermodynamic bias toward Br‐rich nuclei and kinetically frozen halide gradients originate from early‐stage coordination chemistry in moderate‐to‐high bromide compositions. Rational additive selection must therefore consider solvent donicity, Pb–halide binding hierarchy, transient A‐site participation, and the desired intermediate lifetime. Within this framework, phase stability and resistance to light‐induced halide segregation emerge as consequences of controlled intermediate design, positioning additive‐engineered coordination control as a unifying principle for stabilizing Br‐rich WBG perovskites.

## 5 Conclusions and Perspectives

Crystallization intermediates in Br‐rich WBG perovskites are not fixed structural phases, but emergent states encompassing both coordination‐defined species and structural intermediates (e.g., polytypes), governed by the competition among Pb^2+^–halide, Pb^2+^–solvent, Pb^2+^–additive, and hydrogen‐bond‐mediated A‐site interactions. As summarized in Figure 6, these competing interactions define the accessibility and evolution of intermediate configurations during film formation, giving rise to diverse intermediate states, including solvent‐coordinated adducts, polytype‐related structures, and halide‐rich coordination clusters. Within this framework, solvent and additive engineering reshape intermediate pathways by stabilizing, synchronizing, reconstructing, or eliminating intermediate configurations prior to lattice consolidation.

**FIGURE 6:**
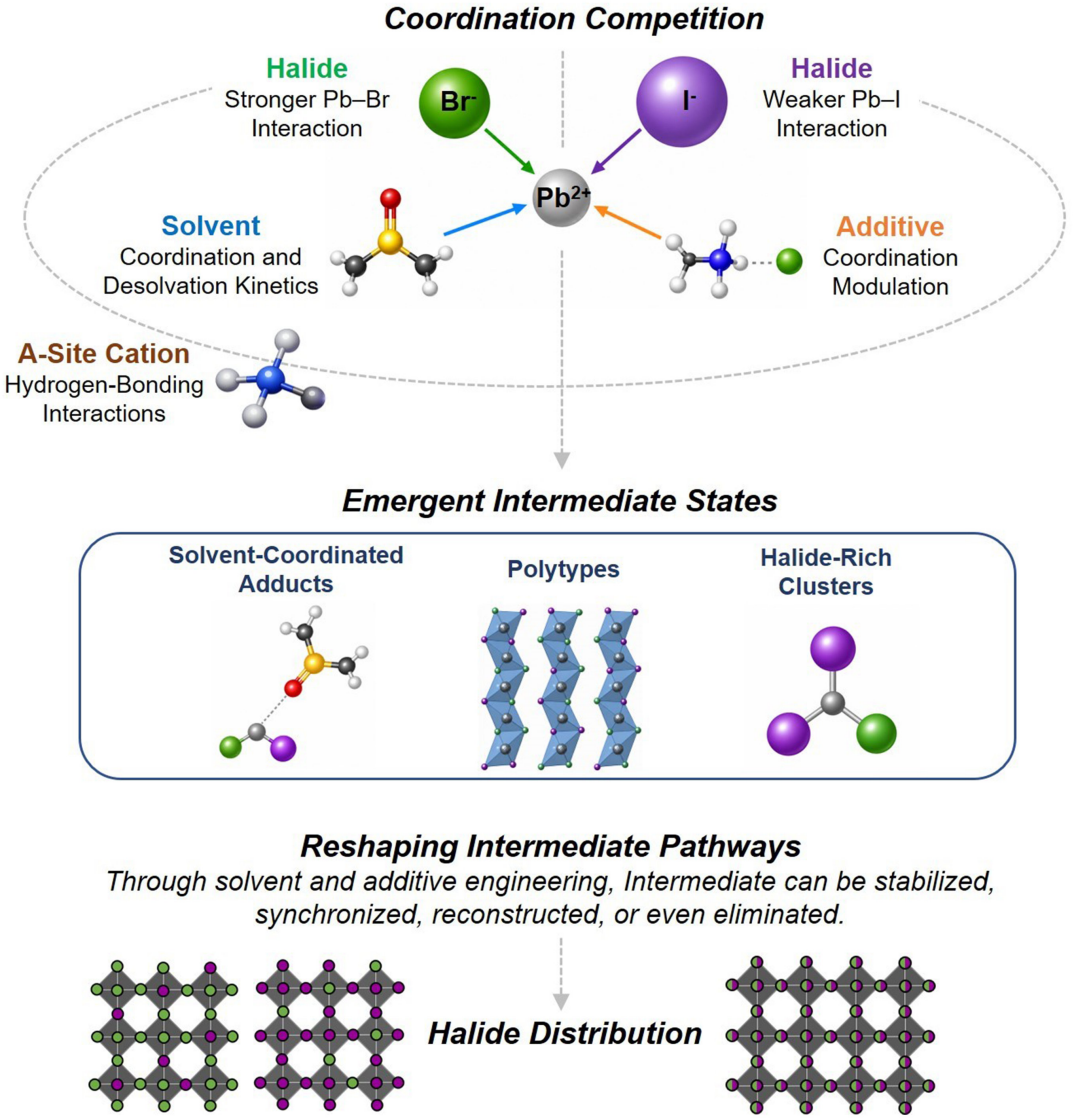
Coordination competition and emergent intermediate states in Br‐rich perovskites. Schematic overview of Pb^2+^‐centered coordination interactions involving halides, solvents, additives, and A‐site cations during crystallization. Representative intermediate states include solvent‐coordinated adducts, polytypes, and halide‐rich clusters, together with their influence on halide distribution.

A unifying descriptor across these strategies is intermediate lifetime. Rapid collapse of intermediates in Br‐rich systems promotes kinetically frozen halide heterogeneity, whereas excessively persistent solvent‐coordinated or polytype‐related states impede phase‐pure cubic growth. Effective crystallization therefore requires synchronized evolution of nucleation, halide redistribution, and lattice assembly. Because Br‐rich compositions exhibit intrinsically strong Pb–Br coordination, subtle variations in precursor chemistry exert amplified effects on intermediate evolution and, consequently, on halide homogeneity and segregation resistance in the final films. Crystallization control in Br‐rich WBG perovskites is thus inseparable from compositional stability control. In application‐relevant contexts—particularly in tandem architectures and IPV systems that rely on stable WBG absorbers—coordination‐centered control over intermediate evolution provides a critical foundation for simultaneously achieving spectral selectivity, compositional homogeneity, and long‐term operational stability.

A more quantitative understanding of the energetics governing intermediate formation and evolution will be important for developing predictive approaches to crystallization control. Table 1 summarizes representative Br‐rich WBG perovskite systems for which energetic information on crystallization intermediates has been reported, together with their compositions and key processing conditions [43, 44, 55, 56]. The available calculations mainly describe specific interactions associated with intermediate stabilization, including solvent–precursor binding and additive–intermediate adsorption, and the reported energetic quantities represent different physical interactions and computational models. Their absolute values therefore cannot be directly compared across studies. Future studies combining calculations of intermediate stability and transformation barriers with experimental measurements of intermediate lifetime and phase‐evolution kinetics could establish more quantitative relationships between intermediate energetics and crystallization pathways.

**TABLE 1:** Representative crystallization intermediates with reported energetic information in Br‐rich WBG perovskites.

**Perovskite composition (*E***_**g**_ **)**	Intermediate	Key processing conditions	Energetic information (DFT)	Reference
FA_0__.8_Cs_0__.15_MA_0__.05_PbI_2__.4_Br_0__.6_ (1.65 eV)	PbI_2_–DMPU	DMF:DMSO = 8:2 (control); DMF:DMSO:DMPU = 8:1.7:0.3 (target)	PbI_2_–solvent binding energy: PbI_2_–DMSO = −27.42; PbI_2_–DMPU = −33.48 kcal mol^−1^	[43]
FA_0__.78_Cs_0__.15_DMA_0__.07_Pb(I_0__.7_Br_0__.3_)_3_ (1.76 eV)	PbX_2_–DMI	DMF:DMSO = 4:1 (control); DMF:DMSO:DMI = 4:0.6:0.4 (target)	PbX_2_–solvent binding energy: PbI_2_–DMSO = −27.78, PbBr_2_–DMSO = −27.97; PbI_2_–DMI = −37.79, PbBr_2_–DMI = −38.16 kcal mol^−1^	[44]
FA_0__.8_Cs_0__.2_PbI_1__.5_Br_1__.5_ (1.86 eV)	PbX_2_ · 2DMSO (X = I, Br)	DMF:DMSO = 6:4; N_2_ gas‐quenching blade coating	PbX_2_ · 2DMSO binding energy: PbI_2_ · 2DMSO, −26.67; PbBr_2_ · 2DMSO, −22.12 kcal mol^−1^	[55]
FA_0__.8_Cs_0__.2_Pb(I_0__.6_Br_0__.4_)_3_ (1.77 eV)	I‐rich 2H	0 mol% ABP (control); 1 mol% ABP (target)	ABP adsorption energy: 2H‐FAPbI_3_ = −0.16; 2H‐FAPbBr_3_ = −0.05 J m^−2^	[56]

Building on such quantitative understanding, machine‐learning approaches may provide a complementary route toward predictive control of crystallization intermediates. Recent studies in broader perovskite systems have demonstrated the use of machine learning to identify additives capable of inducing specific intermediate phases and to extract crystallization‐relevant processing variables from large experimental datasets [57, 58]. Such approaches could be particularly valuable for Br‐rich WBG perovskites, where intermediate evolution is highly sensitive to the coupled effects of halide composition, solvent/additive chemistry, and processing conditions. The resulting multidimensional crystallization landscape makes it challenging to establish simple relationships between processing variables, intermediate stability and lifetime, and final halide homogeneity. Integrating machine learning with in situ characterization and computational descriptors may therefore enable more systematic prediction of intermediate evolution and identification of processing windows that minimize halide heterogeneity in Br‐rich WBG perovskites.

Translating intermediate control to scalable deposition is another important challenge for Br‐rich WBG perovskites, where differences in I^−^ and Br^−^ coordination and rapid crystallization can readily generate compositionally heterogeneous intermediate states. Recent blade‐coating studies demonstrate that controlling solvent‐coordinated or polytype intermediates, or suppressing their crystallization, can mitigate such halide inhomogeneity under scalable processing conditions [55, 56, 59]. These results indicate that intermediate engineering remains relevant beyond laboratory‐scale spin coating, but its implementation must account for the altered solvent‐removal and crystallization kinetics associated with scalable coating. Variations in solvent removal and drying conditions may perturb intermediate evolution and thereby amplify spatial variations in halide distribution across large‐area WBG films. Establishing processing windows that maintain spatially uniform intermediate evolution and halide redistribution will therefore be important for translating intermediate‐control strategies toward large‐area WBG perovskite photovoltaics.

## Funding

This study was supported by the National Research Foundation of Korea (RS‐2023‐00236572 and RS‐2026‐25473161).

## Conflicts of Interest

The author declares no conflicts of interest.

## Data Availability

The data that support the findings of this study are available from the corresponding author upon reasonable request.
